# *Xrcc5/*Ku80 is required for the repair of DNA damage in fully grown meiotically arrested mammalian oocytes

**DOI:** 10.1038/s41419-023-05886-x

**Published:** 2023-07-05

**Authors:** Xuebi Cai, Jessica M. Stringer, Nadeen Zerafa, John Carroll, Karla J. Hutt

**Affiliations:** Department of Anatomy and Developmental Biology, Biomedicine Discovery Institute, Monash University, Clayton, VIC 3800 Australia

**Keywords:** Oogenesis, Meiosis

## Abstract

Mammalian oocytes spend most of their life in a unique state of cell cycle arrest at meiotic prophase I, during which time they are exposed to countless DNA-damaging events. Recent studies have shown that DNA double-strand break repair occurs predominantly via the homologous recombination (HR) pathway in small non-growing meiotically arrested oocytes (primordial follicle stage). However, the DNA repair mechanisms employed by fully grown meiotically arrested oocytes (GV-stage) have not been studied in detail. Here we established a conditional knockout mouse model to explore the role of Ku80, a critical component of the nonhomologous end joining (NHEJ) pathway, in the repair of DNA damage in GV oocytes. GV oocytes lacking Ku80 failed to repair etoposide-induced DNA damage, even when only low levels of damage were sustained. This indicates Ku80 is needed to resolve DSBs and that HR cannot compensate for a compromised NHEJ pathway in fully-grown oocytes. When higher levels of DNA damage were induced, a severe delay in M-phase entry was observed in oocytes lacking XRCC5 compared to wild-type oocytes, suggesting that Ku80-dependent repair of DNA damage is important for the timely release of oocytes from prophase I and resumption of meiosis. Ku80 was also found to be critical for chromosome integrity during meiotic maturation following etoposide exposure. These data demonstrate that Ku80, and NHEJ, are vital for quality control in mammalian GV stage oocytes and reveal that DNA repair pathway choice differs in meiotically arrested oocytes according to growth status.

## Introduction

Mammalian cells encounter thousands of DNA-damaging events on a daily basis, as a consequence of exposure to endogenous and environmental factors [1]. DNA double-strand breaks (DSBs) are the most deleterious form of genetic lesions, as failure to repair, or misrepair of DSBs, can lead to gross chromosomal rearrangements, genomic instability, and even cell death. Homologous recombination (HR) and nonhomologous end-joining (NHEJ) are the two major pathways to repair DSBs and their roles have been well characterized in somatic cells [2]. HR is high fidelity and occurs at the S/G2 phase of the cell cycle as it requires a template for repair. In contrast, NHEJ ligates broken DNA ends and can introduce errors, but can occur at all stages of the cell cycle [3].

In mammals, the majority of oocytes are stored in the ovary in a unique state of prophase I arrest. These oocytes have entered meiosis, progressed through the early stages of the first meiotic prophase then arrested at diplotene. They can remain this way for weeks, months, or years, depending on the species. After birth, some of these oocytes begin to grow and develop but remain arrested at the germinal vesicle (GV)-stage until meiosis I resume. Meiotic resumption occurs in vivo in response to the mid-cycle increase in luteinizing hormone, or on release of the oocyte from the follicle into a suitable culture medium [4]. During meiosis I resumption, oocytes go through germinal vesicle breakdown (GVBD), metaphase I (MI), anaphase I, telophase I, prophase II, and finally arrest at metaphase II (MII), at which stage fertilization can take place [5]. Meiosis II, an initiation of the embryonic cell cycle, is only completed following fertilization. All these complicated events must occur in order for optimal fertility [6].

Female mammals are born with a finite number of oocytes, often referred to as the ovarian reserve, which must sustain fertility throughout the adulthood reproductive lifespan [7]. Ensuring the fidelity of this initial oocyte pool is therefore essential. Throughout their long life, it is a requirement that oocytes efficiently undertake DNA repair, in order to avoid apoptosis, prevent the transmission of genetic errors to the next generation and facilitate reproduction [8, 9]. In mitotic cells, DNA damage triggers a transient G2 arrest, while repair is initiated. In contrast, GV-stage oocytes can resume the meiotic cell cycle even in the presence of extensive DNA damage and are still permitted entry into MI [10–14]. The spindle assembly checkpoint (SAC) is then tasked with protecting the female germline integrity [15–17]. SAC activation following DNA damage triggers a long-lived cell cycle arrest (~25 h) and correlates with a failure to complete the MI to MII transition [13, 18]. Recent studies have begun to address whether these fully grown meiotically mature oocytes have the capacity to mount effective repair, and it has been suggested that the NHEJ pathway in MII-stage is essential in oocytes for limiting the detrimental impact of the occurrence of DNA damage at this late stage of development [19].

NHEJ factors have been identified as important players in DSB repair after irradiation (IR) damage and during V(D)J recombination, including the DNA-end-binding Ku70/Ku80 heterodimer, the protein kinase DNA-PKcs, and the XRCC4/DNA ligase IV complex [20]. These proteins function in three general stages: (1) Ku70/Ku80 heterodimers recognize DSBs and assemble on broken DNA ends where the Ku80 (encoded by *Xrcc5*) C-terminus recruits the catalytic subunit of DNA-PKcs to form holoenzyme DNA-PK [21, 22]. DNA-PK synapses across the break in order to tether the DNA ends [23]; (2) DNA-PKcs then activates the endonuclease activity of Artemis for effective end-processing; and (3) ligation is mediated by the XRCC4/DNA ligase IV complex [24]. Loss of some NHEJ factors, like XRCC4 and DNA ligase IV, results in late embryonic death, whereas loss of *Xrcc5*/Ku80, *Xrcc6*/Ku70, and DNA-PKcs permits viability [25]. While not essential for embryonic development, Ku80 plays a critical role in preventing early senescence and tumorigenesis in multiple species [26–29]. Whether Ku80 plays a role in oocyte development is largely unknown and deserves further attention. In this study, we used a novel mouse model to define the role of Ku80 in mammalian oocytes. Utilizing etoposide treatment to induce DNA damage, we explored the possibility that Ku80 is required for DNA repair and critical determinant of nuclear integrity in oocytes.

## Materials and methods

### Animals

Clustered Regularly Interspaced Short Palindromic Repeats/CRISPR-associated protein 9 and CRISPR (CRISPR/Cas9) genome editing was performed by the Australian Phenomics Network to insert loxP sites flanking exon 4 of *Xrcc5* (Transcript: Xrcc5-201 ENSMUST00000027379.8, exon ENSMUSE00000261283 Supplementary Fig. 1), which we refer to as the floxed (fl) *Xrcc5* allele (*Xrcc5*^*fl*^). Deletion of exon 4 results in the introduction of a premature stop codon and termination of the protein at amino acid 106. Experimental C57BL/6 mice with conditional knockout (cKO) of *Xrcc5* in oocytes and controls were generated by crossing males carrying the Cre recombinase transgene driven by the *Gdf9* promoter [Tg(*Gdf9*-icre)] [30] (*Xrcc5*^*fl/+*^*; Gdf9*^*cre/+*^) with females homozygous for the floxed *Xrcc5* allele (*Xrcc5*^fl/fl^). This mating produced both *Xrcc5* oocyte-cKO mice (*Xrcc5*^*fl/fl*^; *Gdf9*^*cre/+*^) and wildtype littermates (*Xrcc5*^*fl/fl*^*; Gdf9*^*+/+*^) used for experiments. Genotyping was performed by Transnetyx using real-time PCR. All mice were housed under high-barrier conditions with a 12 h light–dark cycle and with free access to mouse chow and water. All animal procedures were compliant with the National Health and Medical Research Council (NHMRC) Australian Code of Practice for the Care and Use of Animals. All animal experiments were approved by the Monash Animal Research Platform Animal Ethics Committee.

### Tissue collection

Ovaries were collected from mice at 6 months of age, fixed in formalin, washed with 70% ethanol, processed and embedded in paraffin before being serially sectioned at 5 μm, and mounted on super frost slides by Monash Histology platform.

### Immunofluorescence staining

Tissue sections were deparaffinized and rehydrated prior to commencing. The primary antibody (anti-XRCC5, NSJ Bioreagents, R31953) was diluted in blocking buffer (5–10% serum/3% bovine serum albumin (BSA)/TN buffer). After overnight incubation with the primary antibody at 4 °C, all tissue sections were washed and incubated with the secondary antibody (Goat anti-Rabbit Alexa 568) at room temperature for 1 h. DAPI staining was performed after the secondary antibody was washed and coverslipping of the slides was then conducted. A Leica SP8 Invert microscope was used for imaging and FIJI software was applied to process and analyze the images.

### Oocyte maturation and stimulation

Stimulation of oocyte maturation was performed via an intraperitoneal (i.p.) injection of pregnant mare serum gonadotropin (5 IU PMSG; Intervet). Ovaries were collected and dissected 44–48 h later. GV-stage oocytes were visualized and collected under a stereoscopic microscope (Leica Wild M8), denuded by mouth-pipetting, and washed in cold PBS. To obtain MII-stage oocytes, 5 IU PMSG (i.p.) was followed 44–48 h later by 5 IU human chorionic gonadotropin (hCG; Intervet) (i.p.). Mice were culled by cervical dislocation 12–16 h after hCG injection. Dilated oviducts were excised and opened with forceps, releasing the cumulus-oocyte complexes (COCs). Then COCs were denuded using 0.3% hyaluronidase (Sigma-Aldrich) in M2 media. The number of ovulated oocytes in each mouse was recorded.

### RT-PCR

**Fig. 1:**
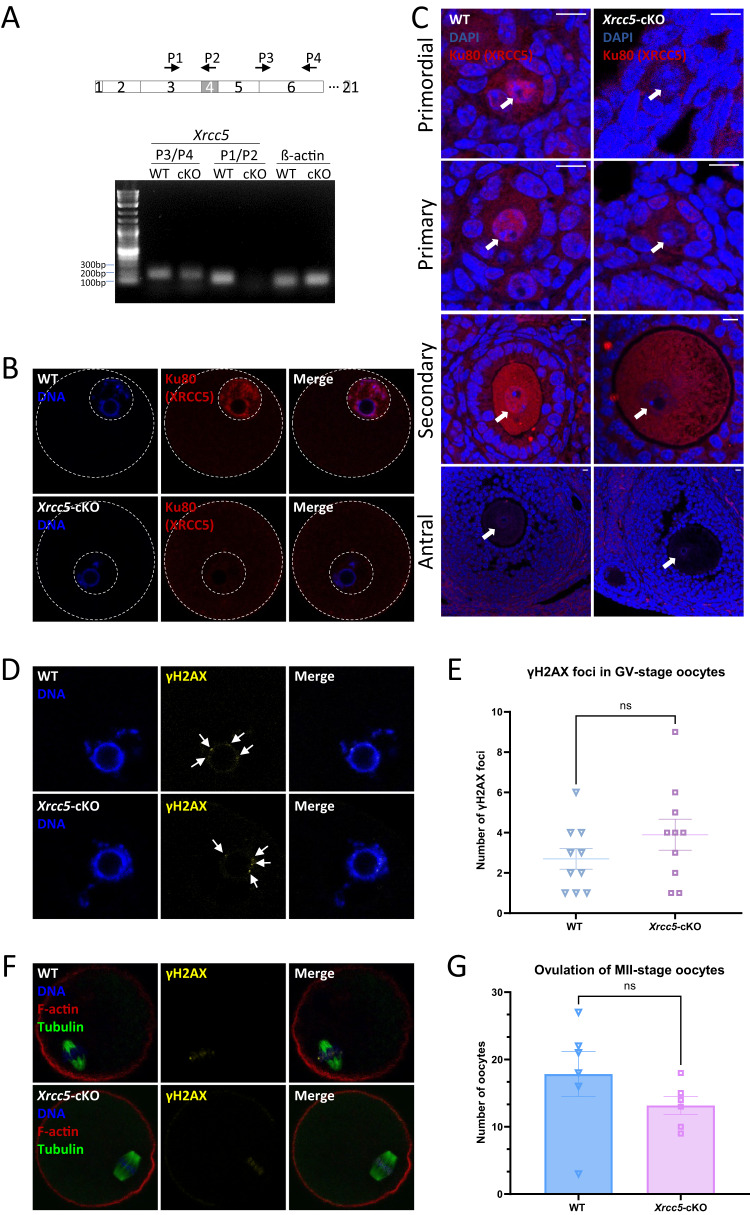
Loss of Ku80 does not increase DNA damage in oocytes, or affect oocyte development and maturation, in mice at 6 months of age. **A** *Xrcc5*-cKO mice were constructed by deleting exon 4 of *xrcc5* gene. To confirm conditional deletion of *Xrcc5* in oocytes, RT-PCR was performed on GV-stage oocytes from wildtype (Xrcc5^fl/fl^; Gdf9^+/+^) and *Xrcc5*-cKO (Xrcc5^fl/fl^; Gdf9^cre/+^) mice (*n* = 3 mice/genotype). ß-actin was used as a loading control. *Xrcc5* gene expression was detected using both pairs of primers in WT oocytes; whereas it was not detected using P1 and P2 in *Xrcc5*-cKO oocytes. **B** Ku80 protein (red) localization in isolated GV oocytes from WT and *Xrcc5*-cKO mice. A white dotted outline indicates the oocyte and nucleus. **C** Ku80 protein (red) localization in ovarian tissue sections from WT (arrows indicate staining in oocyte nuclei) and *Xrcc5*-cKO (arrowheads indicate the absence of staining in oocyte nuclei) mice at 6 months of age. Representative images of primordial, primary, secondary, and antral follicles from top to bottom. Scale bars: 10 µm. **D** Representative images of GV-stage oocytes from 6-month-old WT and *Xrcc5*-cKO mice stained with Hoechst (DNA; blue) and γH2AX (yellow) (*n* = 3 mice/genotype). γH2AX foci are indicated by arrows. **E** Number of γH2AX foci in GV stage oocytes collected from WT and *Xrcc5*-cKO mice at 6-month-old (*n* = 10 oocytes/genotype). **F** Representative images DNA (blue), f-actin (red), αβ-tubulin (green), and γH2AX (yellow) in MII-stage oocytes from WT and *Xrcc5*-cKO mice at 6 months of age (*n* = 10 oocytes/genotype). **G** Number of ovulated MII-stage oocytes collected from WT and XRCC5 cKO mice at 6 months of age (*n* = 6 mice/genotype). Student’s *t*-test was used for statistical analyses (**B** and **D**). Error bars are mean ± SEM, ns no significant difference, *p* > 0.05.

Given the *Xrcc5* oocyte-cKO model was constructed by deleting exon 4 of *Xrcc5* gene, two pairs of primers were designed: P1, 5′-CAGACACCTGATGCTACCAGA-3′ (the forward primer); P2, 5′-GCTGAATCAAATCCATGCACACA-3′ (the reverse primer); P3, 5′-TTTTGCCTTTTCCAATCGAC-3′ (the forward primer); and P4, 5′-CGCCTTCTAAGGACAGCATC-3′ (the reverse primer). P2 is located on exon 4 (Fig. 1A). In accordance with the instructions of Cells-to-CT™ 1-Step Power SYBR™ Green Kit (Cat. # A25599, Invitrogen), 10 GV-stage oocytes were lysed in solution with a simultaneous DNase treatment at room temperature for 5 min. Lysis was terminated via a 2-min incubation with Stop Solution at room temperature. An appropriate amount of RT-PCR Master Mix for the number of reactions was prepared and added to the lysate. RT-PCR was performed using the Agilent Stratagene Mx3000P system (Agilent Technologies). The products were subjected to agarose gel electrophoresis after the standard cycling was finished.

### Etoposide treatment

To induce DSB DNA damage, oocytes were exposed to 5-100 μg/ml of etoposide (E1383, Sigma), in parallel with untreated control (M2 media with vehicle DMSO alone) for 3 h at 38 °C. GV arrest was maintained via the addition of 200 µM 3-isobutyl-1-methylxanthine (IBMX; Sigma-Aldrich) to the M2 medium (Sigma-Aldrich) at a temperature of 38 °C. Following treatment, oocytes were washed in M2 media and either prepared for assessment of DNA damage or for live cell imaging during further maturation.

### Immunofluorescence staining

Oocytes were fixed in 4% paraformaldehyde (PFA) and permeabilized in 1% Triton X-100 solution for 30 min, washed in blocking buffer (1% BSA/phosphate-buffered saline (PBS) + 1/5000 Tween-20), and then blocked at room temperature for 1 h. Subsequently, oocytes were incubated in blocking buffer with primary antibodies (anti-XRCC5 or 1/1000 phospho-histone γH2A.X (Ser139) (Cell Signaling Technology, 9718)) overnight at 4 °C, washed with washing buffer (PBS with 1/1,000 Tween-20 + 1/10,000 Triton X-100), incubated with secondary antibodies, washed again for three times and stained with 1:5000 Hoechst 33342 (Thermo Scientific, 62249) for 10 min and/or with 1/100 anti–β-tubulin (Sigma-Aldrich, T4026) and F-actin 568 (Invitrogen, A-12374) for 1 h. Live cell imaging was performed after oocytes were incubated with 100 nM SiR-DNA (Cat. #CY-SC101, Cytoskeleton, Inc), a green channel DNA probe with an optimal excitation setting of 510 nm and emission setting of 530 nm, for 2 h. A Leica SP8 Invert microscope was used for imaging and FIJI software was applied to process and analyze the images.

### Analysis and statistics

Experiments were replicated 3 times, with 3–6 adult females (as described in figure legends), with individual data points illustrated on graphs. Data were analyzed using GraphPad Prism software (GraphPad Software, Inc., La Jolla, CA, USA) and presented as means ± SEM. Student’s *t*-test or one-way ANOVA followed by Tukey post hoc test was used to analyze normally distributed data, or Kruskal–Wallis for nonparametric data. Differences between groups were considered significant when *P* < 0.05.

## Results

### Ku80 deficiency does not affect oocyte development or maturation

We used mice with a conditional deletion (cKO) of *Xrcc5* in oocytes *(Xrcc5*^*fl/fl*^; *Gdf9*^*cre/+*^) to investigate the role of NHEJ and Ku80-dependent DNA repair during oocyte development and maturation. Mutant mice were confirmed to lack *Xrcc5* mRNA expression in isolated oocytes by RT-PCR (Fig. 1A). Furthermore, immunofluorescent staining revealed that Ku80 protein was present in the nucleus of oocytes from follicles at all developmental stages in tissue sections and isolated oocytes from wildtype (WT) mice (*Xrcc5*^*fl/fl*^; *Gdf9*^*+/+*^), but was absent in *Xrcc5*-cKO mice (Fig. 1B, C).

Others have shown that DNA damage accumulates in oocytes with increasing maternal age [31]. To determine if loss of Ku80 accelerates this age-associated accumulation of DNA damage, fully grown germinal vesicle stage (GVs) and mature (MII) oocytes were collected from 6-month-old WT and *Xrcc5*-cKO mice and stained with DNA damage marker, γH2AX. Very low levels of γH2AX staining were observed in GVs and MIIs from WT and *Xrcc5*-cKO mice (Fig. 1D, F). The number of DSBs, represented by γH2AX foci, show no differences between WT and *Xrcc5*-cKO GV oocytes (Fig. 1E). After superovulation, the number of MII-stage oocytes was similar in WT and *Xrcc5*-cKO mice, and the chromosomes were well-aligned with normal spindle morphologically (Fig. 1F, G). These data suggest that XRCC5 may not be required for oocyte maturation, nor for limiting DNA damage in the oocytes of reproductively older mice.

### Ku80 is crucial to the DNA repair capacity of fully-grown oocytes following induced DNA damage

We next asked if loss of Ku80 impairs the ability of fully grown GV stage oocytes to respond to induced DNA damage. According to our previous work [10], treatment with 5 µg/ml etoposide for 3 h is sufficient to induce DNA damage in somatic cells and germline cells; while higher concentrations block the GV-to-GVBD transition (M phase entry) in oocytes. Thus, in this experiment, GV-stage oocytes from WT and *Xrcc5*-cKO mice were exposed to 0–100 µg/ml etoposide for 3 h, while the meiotic arrest was maintained in IBMX-containing media. Oocytes were then washed in etoposide-free media and DNA damage was assessed at 0, 3, and 6 h post-treatment to test if Ku80 influenced the extent of etoposide-induced DNA damage or the time taken for repair (as determined by loss of γH2AX labeling).

**Fig. 2:**
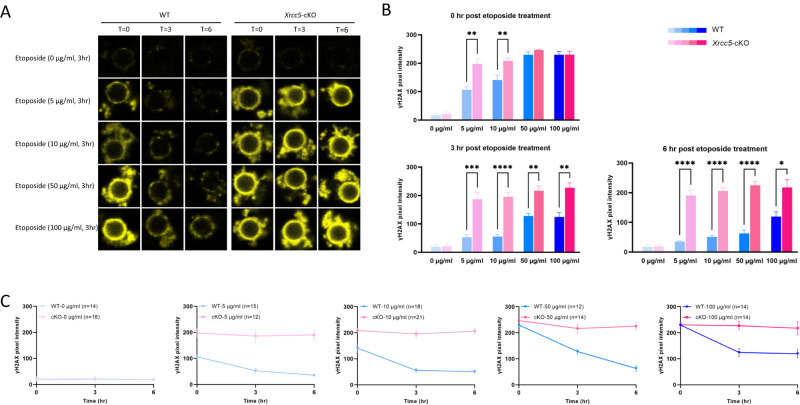
Ku80 is required for the repair of etoposide-induced DNA damage in fully grown GV oocytes. Fully grown GV oocytes were exposed to 0, 5, 10, 50, or 100 µg/ml etoposide for 3 h, then DNA damage and repair were monitored by γH2AX staining. *T* = 0, *T* = 3, and *T* = 6 represent 0, 3, and 6 h post-etoposide treatment, respectively. **A** Representative image of γH2AX staining (yellow). **B** and **C** Quantification of γH2AX pixel intensity. Data were generated from three independent experiments; number of oocytes analyzed is shown in parentheses. Student’s *t*-test was used for statistical analyses (**B** and **C**). Error bars are mean ± SEM, *p < 0.05, **p < 0.01, ***p < 0.001, ****p < 0.0001.

Treatment with all concentrations of etoposide caused DNA damage, as evidenced by the presence of γH2AX (Fig. 2A). Notably, *Xrcc5*-cKO GV oocytes exposed to 5 or 10 µg/ml etoposide showed stronger γH2AX labeling at the end of the initial 3 h (T = 0) exposure when compared to WT ones (Fig. 2B). Furthermore, we observed a reduction in the intensity of γH2AX staining to basal levels in WT oocytes treated with 5–50 µg/ml etoposide over the 6 h recovery period (Fig. 2A, C). Even WT oocytes treated with 100 µg/ml etoposide showed decreased γH2AX intensity at 3 h post-treatment, although no further reduction was observed after this point (Fig. 2B, C). In contrast, γH2AX intensity remained high in *Xrcc5*-cKO oocytes exposed to etoposide. Collectively, these data suggest an indispensable role of Ku80 in the DNA repair capacity of fully grown GV oocytes following etoposide-induced DNA damage.

### Oocyte maturation is severely impaired in *Xrcc5*-cKO oocytes after exposure to 50 µg/ml etoposide

**Fig. 3:**
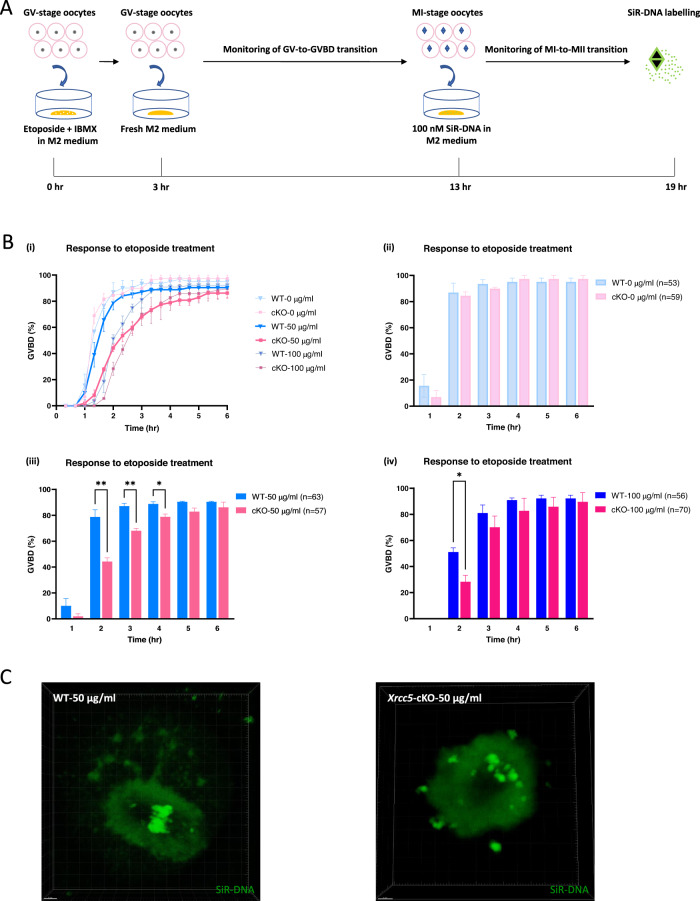
Oocyte maturation is impaired in oocytes from *Xrcc5*-cKO mice following etoposide-induced DNA damage. **A** Fully grown GV oocytes were exposed to 0, 50, or 100 µg/ml etoposide for 3 h, then released from meiotic arrest and in vitro maturation (IVM) monitored. **B** Percentage of oocytes that underwent GVBD transition after etoposide treatment over a 6-h time period. (i) Data were generated from three independent experiments; (ii)–(iv) are drawn from (i); number of oocytes analyzed is shown in parentheses. Student’s *t*-test was used for statistical analyses. Error bars are mean ± SEM, *p < 0.05, **p < 0.01. **C** Representative immunofluorescence images showing the chromosome alignment of WT and *Xrcc5*-cKO oocytes 16 h after treatment with 50 µg/ml etoposide.

In oocytes, a critical step in the resumption of meiosis is the dissolution of the nuclear membrane, referred to as GVBD. Our previous studies have indicated that oocytes can enter M-phase, even in the presence of extensive DNA damage [10]. Given this information, we next sought to address whether the timing and rate of the GV-to-GVBD transition would be influenced by the deletion of *Xrcc5*. For this purpose, WT and *Xrcc5*-cKO GV oocytes were maintained in IBMX for 3 h and exposed to 0 (no exposure), 5, 10, 50, or 100 µg/ml etoposide. Oocytes were then released from arrest and live cell imaging was conducted at 5-minute intervals (Fig. 3A, Supplementary movies 1–8, and 11, 12).

In WT and *Xrcc5*-cKO oocytes treated with 5 or 10 µg/ml etoposide, the timing, and rate of GV-to-GVBD transition were similar to that observed in untreated oocytes, and the chromosomes aligned normally on the MI spindle (Supplementary movies 1–6; Supplementary Fig. 2). A failure to complete the MI to MII transition was observed in almost all treated oocytes, while 70% of untreated oocytes were able to enter MII stage (Supplementary Fig. 3). In oocytes treated with 50 µg/ml etoposide, the timing of GV-to-GVBD transition was severely delayed in the *Xrcc5*-cKO groups when compared to WT groups, but the percentage of oocytes that made it to GVBD was similar between the two groups by 6 h post-released from IBMX (Fig. 3B; Supplementary movies 7, 8). On labeling of DNA in oocytes treated with 50 µg/ml etoposide, disrupted chromosome alignment was observed in all *Xrcc5*-cKO MI oocytes, but not WT MI oocytes (Fig. 3C; Supplementary movies 9, 10). These findings indicate that Ku80 plays a critical role in repairing DNA double-strand breaks and possibly holding the damaged DNA together in fully grown oocytes, consistent with its established roles in somatic cells.

### Ku80 is essential for oocyte integrity after exposure to 100 µg/ml etoposide

**Fig. 4:**
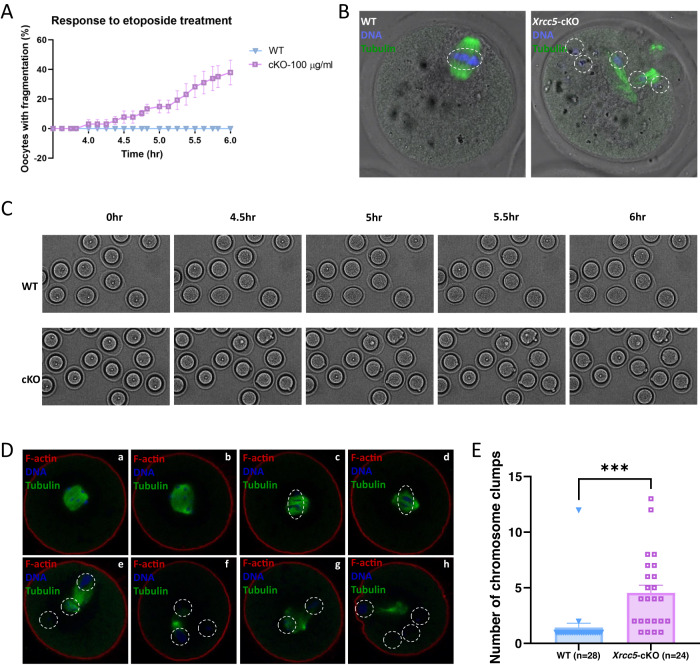
Loss of Ku80 is associated with loss of chromosome integrity and oocyte fragmentation following treatment with 100 µg/ml etoposide. **A** Percentage of WT and *Xrcc5*-cKO oocytes with fragmentation relative to time post-released from IBMX. **B** Representative images of WT and *Xrcc5*-cKO oocytes treated with 100 µg/ml etoposide. Hoechst was used to label the DNA (blue) on the metaphase plate, and αβ-tubulin (green) was utilized to label the spindle. White dotted line circles were used to mark the chromosome clumps. **C** Representative images showing the occurrence of DNA fragmentation with time post-released from IBMX. GV-stage oocytes were harvested from 3 mice/genotype. **D** Representative images of oocytes 6 h post-released from IBMX. (a) untreated WT oocyte; (b) untreated KO oocyte; (c) and (d) 100 µg/ml etoposide-treated WT oocytes; (e)–(h) 100 µg/ml etoposide-treated KO oocytes. Hoechst was used to label the DNA (blue) on the metaphase plate, f-actin (red) was applied to mark the oolemma, and αβ-tubulin (green) was utilized to label the spindle. White dotted line circles were used to mark the chromosome clumps. **E** Number of chromosome clumps in WT and *Xrcc5*-cKO oocytes. Data were generated from three independent experiments; number of oocytes analyzed is shown in parentheses. Student’s *t*-test was used for statistical analyses. Error bars are mean ± SEM, ***p < 0.001.

During live-cell imaging under bright field conditions, WT and *Xrcc5*-cKO oocytes treated with 0, 5, 10, and 50 µg/ml etoposide showed normal morphology following release from IMBX (Supplementary movies 1–8). In contrast, although normal morphology was maintained in WT oocytes treated with 100 µg/ml etoposide, oocyte fragmentation was observed at 4 h in *Xrcc5*-cKO oocytes (Supplementary movies 11, 12). The percentage of *Xrcc5*-cKO oocytes with fragmentation reached a peak 6 h post-released from IBMX (Fig. 4A, C). We also noted a slightly delayed timing of GV-to-GVBD transition in *Xrcc5*-cKO oocytes treated with 100 µg/ml etoposide relative to WT (Fig. 3B; Supplementary movies 11, 12). These oocytes were fixed and stained at the end of incubation (16 h post-released from IBMX). Interestingly, chromosomes were aligned well in the middle of the spindle in WT oocytes but were scattered in *Xrcc5*-cKO oocytes (Fig. 4B).

Considering most oocyte fragments had broken down before reaching the end of incubation (Supplementary movies 11, 12), oocytes were subsequently analyzed at 6 h post-released from IBMX, when the percentage of cell fragmentation reached the peak. Compared to the untreated oocytes, chromosomes were aggregated in WT oocytes treated with 100 µg/ml etoposide (Fig. 4D). In *Xrcc5*-cKO oocytes, chromosomes were scattered, with or without visible oocyte fragments, after exposure to 100 µg/ml etoposide, with an average of 4.5 chromosome clumps present (Fig. 4E).

## Discussion

Efficient DNA repair is critical for fertility in all sexually reproducing eukaryotes. Meiotic recombination in germ cells is believed to have evolved from somatic DNA repair mechanisms, and both mismatch repair (MMR) and HR pathways are essential for this process [32]. HR has been shown to be the dominant mechanism of DNA repair in prophase I-arrested primordial follicle oocytes exposed to genotoxic agents, and appears to be largely responsible for protecting this pool of follicles from DNA damage during their long lifespan [33]. In fully grown GV-stage and preovulatory MII-stage oocytes, the ability to detect physiological DNA damage remains high, but the establishment of an effective DNA damage response sufficient to induce a cell cycle checkpoint-mediated arrest is reported to be reserved only for very severe DNA damage, for instance, that induced by high concentrations of etoposide or neocarzinostatin [13, 34]. Repair mechanisms have not been investigated extensively in fully grown oocytes, although recent observations showing inhibitors of DNA-PKcs and DNA ligase IV prevent repair after etoposide treatment, suggest the NHEJ pathway is active in mouse oocytes at the MII stage [19]. In this study, we use oocyte-specific genetic ablation of *Xrcc5* to demonstrate important roles for HNEJ, and Ku80 in particular, for the repair of DNA double-strand breaks in fully grown GV stage oocytes.

Mice completely lacking Ku80 are fertile and their offspring viable [35], which is consistent with our finding that *Xrcc5*-cKO mice exhibited normal oocyte development and maturation. However, mice with homozygous defects in *Xrcc5* have a slightly earlier onset of cancer [36] and, in humans, a rare microsatellite polymorphism in Ku80 is associated with cancer of varying radiosensitivity. These observations suggest that Ku80 plays an important role in repairing a large extent of accumulated DNA damage rather than simply ‘physiological’ DNA DSBs [35]. Hence, we challenged oocytes from WT and *Xrcc5*-cKO mice with etoposide, a topoisomerase II inhibitor that can cause DNA DSBs in both somatic cells and germline cells [10, 37–40]. Treatment with etoposide (5 μg/ml for 3 h) has previously been shown to cause DNA damage in oocytes, as evidenced by the presence of γH2AX staining [41]. Consistent with these earlier observations, etoposide elicited a significant, dose-dependent increase in γH2AX staining, and DNA DSBs, in WT and *Xrcc5*-cKO GV-stage mouse oocytes. GV-stage oocytes expressing Ku80 were capable of mounting an effective DNA damage repair response, evidenced by a gradual decline in the γH2AX expression at DSB sites (loss of γH2AX signal at DSB sites is widely acknowledged to reflect the completion of repair of DNA DSBs [42, 43]). In contrast, in the absence of Ku80, γH2AX foci were not resolved in a timely manner and expression remained at elevated levels. These data suggest an indispensable role for Ku80, and NHEJ, in the DNA repair capacity of DSBs in fully grown meiotically arrested GV stage oocytes. Moreover, although HR may play a role in the repair of DSBs in oocytes, these data further imply that HR cannot compensate for a compromised NHEJ pathway in fully grown oocytes, or, alternatively, it may be that HR pathway is saturated by the high levels of damage induced in this experimental paradigm Interestingly, non-growing prophase arrested oocytes from primordial follicles appear to preferentially utilize HR to repair DSBs [33]. It is unclear why immature and fully grown oocytes show differences in repair pathway choice, despite both oocyte classes being in the same stage of meiotic arrest, albeit with potentially different chromatin structures. One possible explanation is that in primordial follicles, chromosomes may be perfectly aligned to ensure that HR is effective, whereas, in fully grown GV oocytes, this alignment is less stringent as the synaptonemal complexes start to dissolve. Additionally, given evolution of DNA repair pathways has probably been driven to repair stochastic DNA damage events rather than the en masse damage induced by exposure to genotoxic agents such as etoposide, the relative roles of HR and NHEJ in DNA damage repair in oocytes needs further investigation, including in response to the ‘physiological’ induction of DNA damage. For example, similar studies to those reported here could be undertaken using oocytes deficient in the HR, by using *Rad51*-cKO oocytes or Rad51 inhibitors, alone and in combination with DNA-PKcs inhibitors, to further investigate the relative roles of HR and NHEJ.

We and others have shown that in GV-stage oocytes, etoposide delays, but does not prevent entry into M-phase [13, 34]. This accords with our findings that the rates of GV-to-GVBD transition in WT and *Xrcc5*-cKO groups treated with etoposide were almost equal to those in untreated ones. Indeed, despite the presence of DNA damage after the treatment of etoposide at low concentration (5–10 μg/ml, 3 h), oocytes were able to undergo GVBD and enter the M phase at near normal kinetics. As the etoposide concentrations increased (50–100 μg/ml, 3 h), the ability to undergo GVBD gradually reduced. Notably, *Xrcc5*-cKO oocytes treated with high concentrations of etoposide (50–100 μg/ml) experienced a more severe delay in GV-to-GVBD transition compared to WT oocytes. A previous study has reported that delayed M-phase entry is positively correlated with the extent of DNA damage [11], suggesting that the extent of damage occurring in the absence of Ku80 might be increased due to the inability to undertake NHEJ repair. Collectively, these observations indicate Ku80-mediated DNA repair is important for the timely entry into the M phase.

Despite encountering severe DNA lesions and prolonged arrest, oocytes retained the ability to enter M-phase, albeit more slowly, carrying with them the potential for chromosomal aberrations. In the presence of Ku80, chromosomes aligned on the MI metaphase plate but consistent with previous findings all oocytes arrested without extruding the first polar body. In WT oocytes chromosome alignment occurred even when exposed to high concentrations of etoposide (50–100 μg/ml) for 3 h. However, in the absence of Ku80, chromosomes typically scattered in clumps and were associated with disorganized microtubules. These data suggest that Ku80 is critical for the integrity of oocyte DNA and its ability to organize a functional MI spindle, which is essential for reproductive success [31, 44, 45]. Given Ku80 can recruit DNA-PK to tether the broken ends of the DNA [23], it is not surprising that the chromosomes cannot align in the absence of Ku80. In ovarian cancer cells (A4-T), irregular chromosomal segregation also occurred when silencing RAD50 and XRCC5, under conditions of genomic stress [46]. Other supportive evidence for the role of XRCC5 in chromosome integrity was also shown in mouse somatic cells deficient for XRCC5, which displayed a marked increase in chromosomal aberrations, including translocations, aneuploidy, chromatid interchanges, and breaks [36].

To conclude, we established a conditional knockout mouse model to study the role of XRCC5 in mammalian oocytes. Analysis of the oocyte DNA damage response suggests that XRCC5 is required for efficient DNA repair when exposed to genotoxic insults. Loss of XRCC5 leads to a failure in DNA damage recovery, even when only low levels of damage are sustained, and a severe delay in the entry to M-phase when exposed to higher levels of DNA damage. Moreover, our findings provide the first evidence that XRCC5 is critical for chromosome integrity during oocyte maturation, extending the current knowledge of the quality control mechanisms in fully grown oocytes. This work encourages a reappraisal of the long-held paradigm that fully grown oocytes possess relatively inefficient DNA damage response mechanisms and are largely refractory to DNA repair. Given the modern environment is releasing more genotoxic insults as an inevitable result of social and economic development, which makes people suffer from more DNA lesions on a daily basis, keeping the genetic integrity of XRCC5 and other DNA repair genes is of great importance for safeguarding female fertility.

## Supplementary information

Supplementary figure and movie legends

Supplementary Figure 1

Supplementary Figure 2

Supplementary Figure 3

Supplementary movie 1

Supplementary movie 2

Supplementary movie 3

Supplementary movie 4

Supplementary movie 5

Supplementary movie 6

Supplementary movie 7

Supplementary movie 8

Supplementary movie 9

Supplementary movie 10

Supplementary movie 11

Supplementary movie 12

Reproducibility checklist

## Data Availability

All data related to the manuscript can be made available upon request.
