# Supplementary material for: Xrcc5/Ku80 is required for the repair of DNA damage in fully grown meiotically arrested mammalian oocytes
Source: Cell Death Dis. 2023 Jul 5;14(7):397. doi: 10.1038/s41419-023-05886-x (PMC10322932; doi:10.1038/s41419-023-05886-x)
Supplement: Supplementary file 1 — Supplementary figure and movie legends [file 41419_2023_5886_MOESM1_ESM.docx]

**Supplementary Information**

**Title: Xrcc5/Ku80 is required for the repair of DNA damage in fully grown meiotically arrested mammalian oocytes**

Xuebi Cai^1^, Jessica M Stringer^1^, Nadeen Zerafa^1^, John Carroll^1^, Karla J Hutt^1*^

^1^Biomedicine Discovery Institute, Department of Anatomy and Developmental Biology, Monash University, Clayton, VIC 3800, Australia.

*Correspondence to: [karla.hutt@monash.edu](mailto:karla.hutt@monash.edu)

**Supplementary figure legends**

**Supplementary figure 1: Repair template that was used for making the Xrcc5 CKO mouse (809bp)**

**Supplementary figure 2: Representative images showing chromosome alignment in oocytes treated with 10 µg/ml etoposide.** SiR-DNA was used to label DNA (green). Chromosomes were aligned well in both WT and *Xrcc5*-cKO oocytes. N=3 independent experiments; n >50 oocytes per group. See supplementary movies 5 and 6 for time-lapse movies related to these data.

**Supplementary figure 3: Percentage of oocytes undergoing MI-to-MII transition.** ~70% of untreated oocytes from both WT and *Xrcc5*-cKO groups were able to enter MII. In contrast, very few etoposide (5 µg/ml, 10 µg/ml) treated oocytes were able to enter MII. N=3 independent experiments; n >50 oocytes per group. Student’s t test was used for statistical analysis. Error bars are mean ± SEM, ns=no significant difference. See supplementary movies 1-6 for time-lapse movies related to these data.

**Supplementary movie legends**

**Supplementary movie 1:** Representative movie showing *in vitro* maturation of untreated WT oocytes. N=3 independent experiments; n >50 oocytes per group.

**Supplementary movie 2:** Representative movie showing *in vitro* maturation of untreated *Xrcc5*-cKO oocytes. N=3 independent experiments; n >50 oocytes per group.

**Supplementary movie 3:** Representative movie showing *in vitro* maturation of WT oocytes treated with 5 µg/ml etoposide. N=3 independent experiments; n >50 oocytes per group.

**Supplementary movie 4:** Representative movie showing *in vitro* maturation of *Xrcc5*-cKO oocytes treated with 5 µg/ml etoposide. N=3 independent experiments; n >50 oocytes per group.

**Supplementary movie 5:** Representative movie showing *in vitro* maturation of WT oocytes treated with 10 µg/ml etoposide. N=3 independent experiments; n >50 oocytes per group.

**Supplementary movie 6:** Representative movie showing *in vitro* maturation of *Xrcc5*-cKO oocytes treated with 10 µg/ml etoposide. N=3 independent experiments; n >50 oocytes per group.

**Supplementary movie 7:** Representative movie showing *in vitro* maturation of WT oocytes treated with 50 µg/ml etoposide. N=3 independent experiments; n >50 oocytes per group.

**Supplementary movie 8:** Representative movie showing *in vitro* maturation of *Xrcc5*-cKO oocytes treated with 50 µg/ml etoposide. N=3 independent experiments; n >50 oocytes per group.

**Supplementary movie 9:** Three-dimensional (3D) movie showing the alignment of chromosomes in WT oocytes treated with 50 µg/ml etoposide. N=3 independent experiments; n >50 oocytes per group.

**Supplementary movie 10:** Three-dimensional (3D) movie showing the alignment of chromosomes in *Xrcc5*-cKO oocytes treated with 50 µg/ml etoposide. N=3 independent experiments; n >50 oocytes per group.

**Supplementary movie 11:** Representative movie showing *in vitro* maturation of WT oocytes treated with 100 µg/ml etoposide. N=3 independent experiments; n >50 oocytes per group.

**Supplementary movie 12:** Representative movie showing *in vitro* maturation of *Xrcc5*-cKO oocytes treated with 100 µg/ml etoposide. N=3 independent experiments; n >50 oocytes per group.
