# Supplementary material for: Xrcc5/Ku80 is required for the repair of DNA damage in fully grown meiotically arrested mammalian oocytes
Source: Cell Death Dis. 2023 Jul 5;14(7):397. doi: 10.1038/s41419-023-05886-x (PMC10322932; doi:10.1038/s41419-023-05886-x)
Supplement: Supplementary file 2 — Supplementary Figure 1 [file 41419_2023_5886_MOESM2_ESM.pdf]

Supplemental data 1. Repair template that was used for making the Xrcc5 CKO mouse (809 bp)

LoxP
Exon Xrcc5

CCCTGTCTCAACACACACACACAGAGAATAGGGTTCTAATCTTTAGTATTTTTCTAAAAAATTAGTAG
TACCATTCTTGACCACTAGGAGGTGCTGCTGTGTTTGGTGTTGCTTGCTGGCTTGCATTGTATTTAAG
AGTGGATTGCTGACGAAATATATAACTTCGTATAGCATACATTATACGAAGTTATGGGTTCTAAGAA
TTGTTAAATGACATCTTAGAGTTTTTGTTAAATGGATATGTGTTTTTTCCTCCACACTATTTGTTAGGA
TGTATATAAAGCACTTATGAAATAGAGTCTGGGACACATGGGGCAAGCCCTTTTAAGTTGTCCAGTG
TCTGCTGTTGTCAGTAGTATGGGCTTTGCTGGGAGAAACTGTGATGTTCATGGTTAAGAAGGGAGT
GTTGGTGTTCTTTCAATCCTTAACTAGCTGAGTCCTGGACGCCCTGATTGTGTGCATGGATTTGATTC
AGCGTGAAACCATGTGAGTGTCTCACCTGAAGACAGGAGTGGGGCTTTCTGGGAAAAACAGGGGT
CCATGTGTTCAGAGGATGCGTGGTTTGTTGTTAATTAAGTAGCGCGTTAGTCTGTCAGCGCCTTTCTA
GCTTATGAAAGTTTAAAACAAAGGACTACCTTTGGTGGGAGTTTCTCATAACTTCGTATAGCATACAT
TATACGAAGTTATTGGATGAAATAAGTATTTGGCATACTTTGGTTTTCTTGTTAACTAGCTTAAACTA
GTTAACCCCAAATCTTTAAAAAGTACCAAGTGGTATGATCCATAAGTAGTTGGTGTGCTGTACACAG
