# Supplementary figures and images for: Xrcc5/Ku80 is required for the repair of DNA damage in fully grown meiotically arrested mammalian oocytes
Source: Cell Death Dis. 2023 Jul 5;14(7):397. doi: 10.1038/s41419-023-05886-x (PMC10322932; doi:10.1038/s41419-023-05886-x)

WT

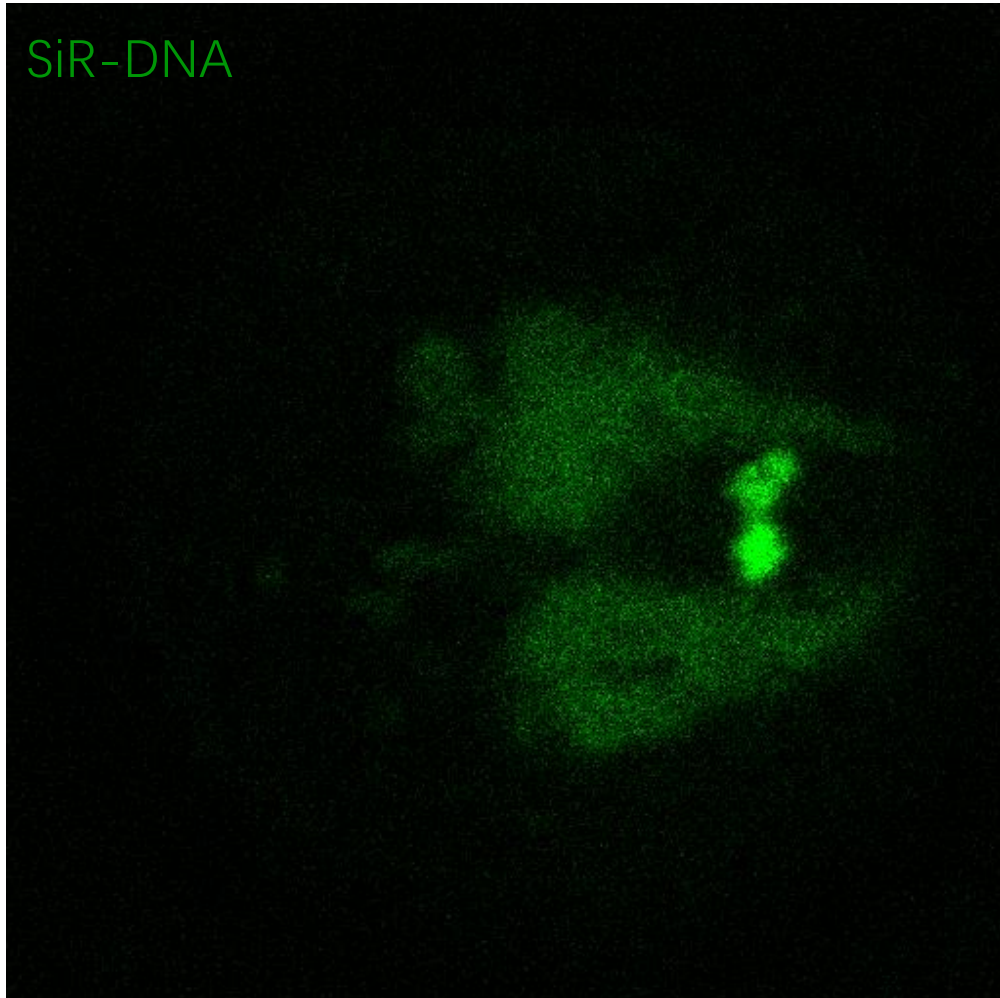

*Xrcc5*-cKO

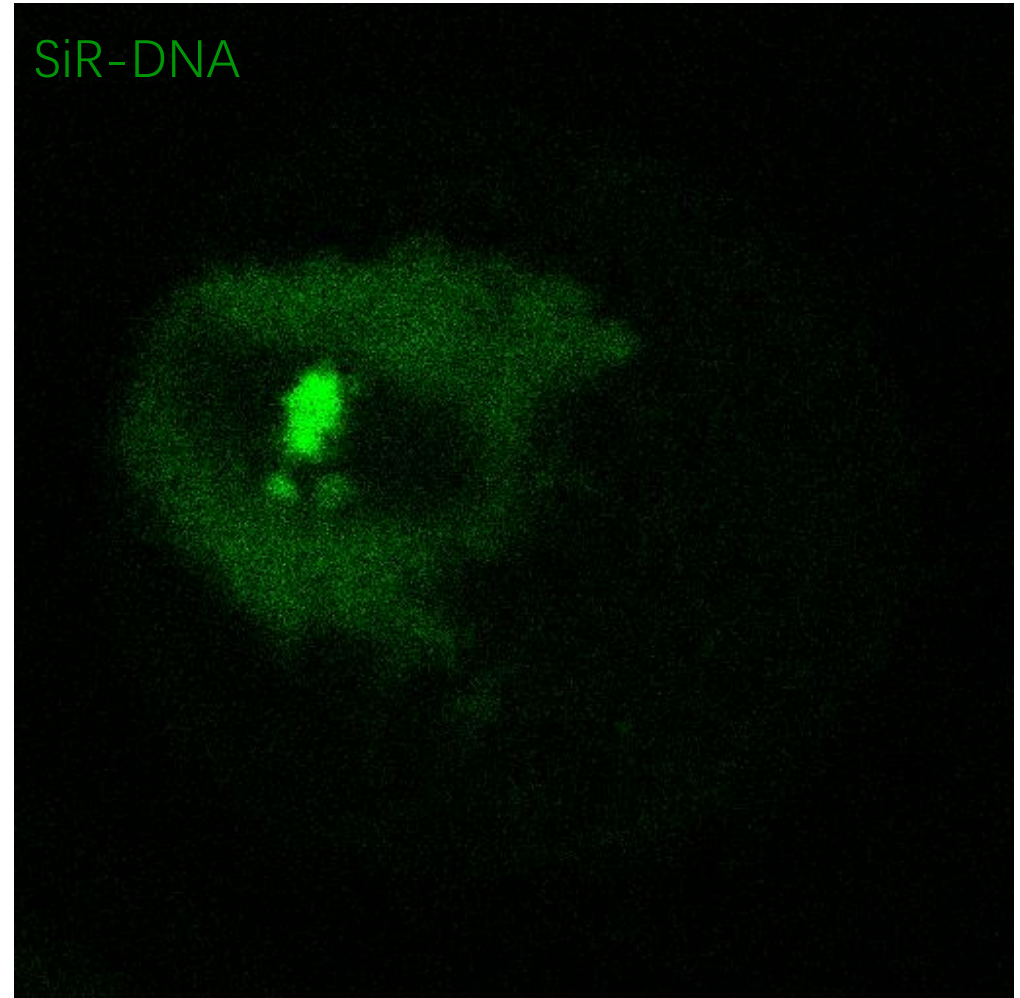

Supplement: Supplementary file 3 — Supplementary Figure 2 [file 41419_2023_5886_MOESM3_ESM.pdf]

## MI-to-MII transition

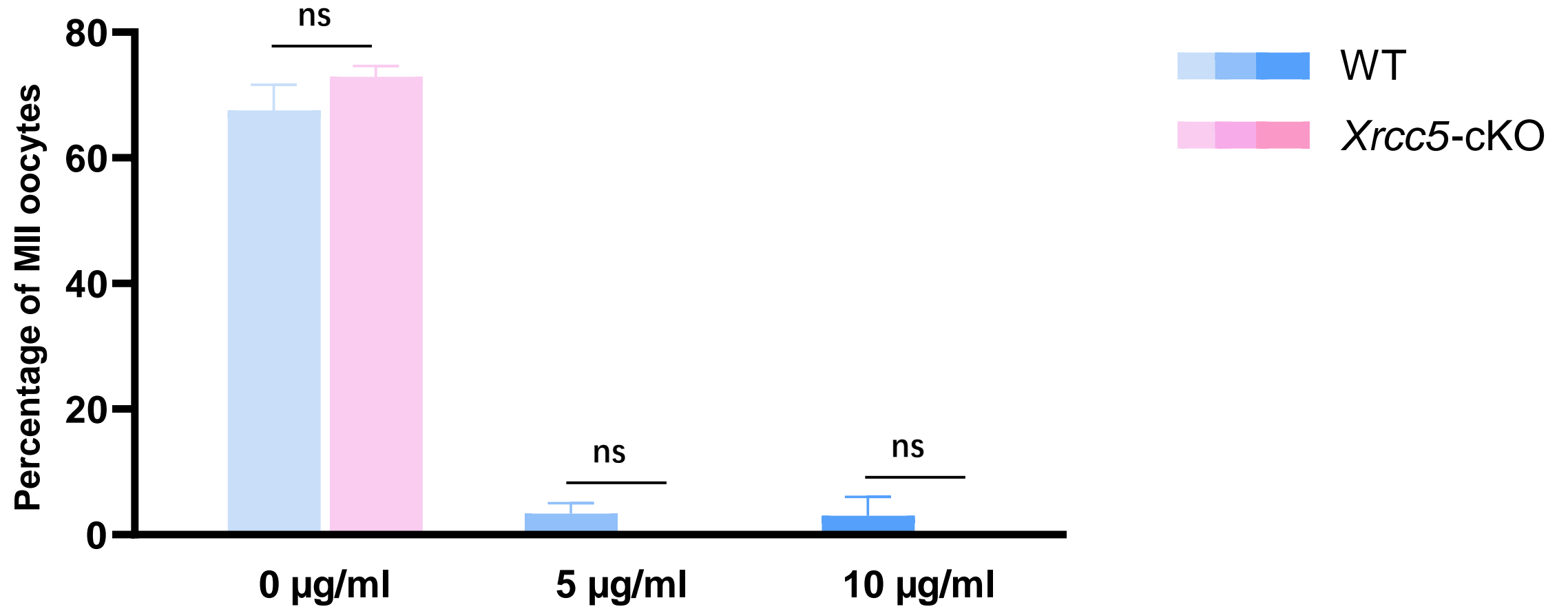

Supplement: Supplementary file 4 — Supplementary Figure 3 [file 41419_2023_5886_MOESM4_ESM.pdf]
